# AO Spine/Praxis Clinical Practice Guidelines for the Management of Acute Spinal Cord Injury: An Introduction to a Focus Issue

**DOI:** 10.1177/21925682231189928

**Published:** 2024-03-25

**Authors:** Brian K. Kwon, Lindsay A. Tetreault, Nathan Evaniew, Andrea C. Skelly, Michael G. Fehlings

**Affiliations:** 1 Department of Orthopaedics, 8166 University of British Columbia, Vancouver, BC, Canada; 2 International Collaboration on Repair Discoveries (ICORD), University of British Columbia, Vancouver, BC, Canada; 3 Department of Neurology, 12297 NYU Langone Medical Center, New York, NY, USA; 4 Department of Surgery, McCaig Institute for Bone and Joint Health, Orthopaedic Surgery, Cumming School of Medicine, 2129 University of Calgary, Calgary, AB, Canada; 5 Aggregate Analytics, Inc., Fircrest, WA, USA; 6 Division of Neurosurgery and Spine Program, Department of Surgery, University of Toronto, Toronto, ON, Canada; 7 Division of Neurosurgery, Krembil Neuroscience Centre, Toronto Western Hospital, 7989 University Health Network, Toronto, ON, Canada

**Keywords:** acute spinal cord injury, clinical practice guideline, recommendations, surgical decompression, intra-operative spinal cord injury, hemodynamic management, intra-operative neuromonitoring

## Abstract

**Study Design:**

Narrative overview and summary.

**Objectives:**

The objective of this introductory manuscript is to provide an overview of the effort that was undertaken to establish clinical practice guidelines for a number of important topics in spinal cord injury (SCI). These topics included: 1. The role and timing of surgical decompression after acute traumatic SCI; 2. The hemodynamic management of acute traumatic SCI; and 3. The definition, diagnosis, and management of intra-operative SCI. Here, we introduce the rationale for the guidelines, the methodology utilized, and summarize how the topics are addressed within various manuscripts of this Focus Issue.

**Methods:**

The key clinical questions were defined using the PICO format for treatment reviews (patient; intervention; comparison; outcomes) or PPO format (patient, prognostic factor, outcomes) for risk factor review. Multi-disciplinary, international guideline development groups (GDGs) were established to evaluate and collate the available evidence in a rigorous, systematic manner, followed by a review of systematically obtained evidence within the framework of the Grades of Recommendation, Assessment, Development, and Evaluation (GRADE) criteria and application of the Evidence to Decision process. Consensus meetings, using a modified Delphi approach, were held with the multidisciplinary, international GDGs using online video-conferencing technology and anonymous voting to develop the final recommendations for each of the topics addressed. All systematic review protocols followed PRISMA standards and were registered on PROSPERO; all potential conflicts were vetted in an open and transparent manner. The funders (AO Spine and Praxis Spinal Cord Institute) had no influence over editorial content or the guidelines process).

**Results:**

Updated guidelines were established for the timing of surgical decompression after acute SCI, with surgical decompression within 24 hours of injury now “recommended” as a treatment option. Updated guidelines were also established for hemodynamic management, with an expanded target range for mean arterial pressure (MAP) of 75-80 to 90-95 mmHg for between 3 to 7 days post-injury now “suggested” as a treatment option. The available literature mandated scoping and systematic reviews on the topic of intra-operative SCI, and this resulted in manuscripts to address the definition, frequency, and risk factors, to define the role of intra-operative neuromonitoring, and to suggest an evidence-based care pathway for management.

**Conclusion:**

A rigorous process following GRADE standards was undertaken to review the available evidence and establish guideline recommendations around the role and timing of surgery in acute SCI, optimal hemodynamic management of acute SCI and the prevention, diagnosis and management of intraoperative SCI. This effort also identified key knowledge gaps and future directions for study, which will serve to refine these recommendations in the future.

## Introduction

Suffering an acute spinal cord injury (SCI) is a catastrophic event and remains a great challenge for biomedical science; one that has stimulated and stymied decades of global research effort in search of effective treatment options to minimize or reverse paralysis and improve neurological outcome. Progress has undeniably been made in the medical, surgical, and rehabilitative care of SCI. The field strives to optimize these available clinical approaches whilst developing other novel treatment approaches such as pharmacologic, biologic and bioengineering therapies.^1^ There is much optimism in the field around the emergence of such novel treatment approaches; but in the meantime, clinicians who manage these patients are faced with the difficulty of *“doing the best they can”* to optimize neurological outcome.

This difficulty arises from the fact that it has been quite challenging for our field to demonstrate the extent to which our current clinical approaches to SCI improve neurological outcome. On one hand, while it may seem totally obvious that decompressing the traumatically injured and persistently compressed spinal cord as soon as possible would be beneficial (and many animal studies support this contention^2^), actually demonstrating this benefit of early surgical decompression has been far from straightforward in human SCI over the past few decades. Similarly, while it seems intuitive that the traumatically injured spinal cord with its disrupted microvasculature^3^ should have its perfusion supported through mean arterial pressure (MAP) augmentation, determining how to best do this to minimize ischemic secondary injury has not been well established (although recommendations around this have been circulating for decades). Finally, in instances where injury to the spinal cord occurs intra-operatively (as evidenced by changes in neurophysiologic monitoring or post-operative examination^4,5^), guidance about what immediate measures to institute in an effort to improve function is desperately sought by any spine surgeon who has found themself in this situation *(and most of us unfortunately have)*.

For these 3 issues - the timing of surgical decompression, the optimization of spinal cord perfusion, and the management of intra-operative SCI (ISCI) - one of the fundamental problems is the heterogeneity of SCI and resultant variability in how people recover neurologically after injury. And of course, the relatively low incidence (particularly for ISCI) is problematic, as it makes it difficult to accrue large clinical experience and or evidence from study cohorts. Thus, definitively demonstrating that a particular treatment approach leads to better neurological recovery when such variability in recovery exists has been challenging, and clinicians are left seeking guidance around what the best currently available evidence suggests they ought to do in the moment.

To this end, clinical practice guidelines can play an important role in evaluating and summarizing the available evidence, and then contextualizing it around the decision-making framework that clinicians are accustomed to working within on a daily basis: considering the strength of the available evidence, weighing the balance of risks and benefits, leaning on clinical experience/judgment, considering stakeholder perspectives, and factoring in the health care environment that they exist within. Here, we undertook the development of guidelines around these 3 important clinical issues: the timing of surgical decompression, the hemodynamic management of SCI, as well as the identification and management of intra-operative SCI. For the timing of surgical decompression, we felt that an update of the 2017 AO Spine Guideline on this topic^6^ was warranted, given the evidence that had emerged since its publication. In particular, the paucity of evidence and inability to perform a meta-analysis on the role of early surgical decompression precluded a strong recommendation and thus early surgical decompression within 24 hours post-injury was only a “suggested” treatment option.^6^ For hemodynamic management, we acknowledged that many papers had been published since the 2013 AANS/CNS guidelines on this topic^7^ and felt that an update was warranted. And for intra-operative SCI, based on an international survey of key stakeholders^8^ and discussions in the AO Spine Knowledge Forums, we felt that a formal guideline would be valuable, particularly given the immense gravity of this problem for the surgeon and patient facing it, even if it were a rare event.

From a methodologic standpoint, we endeavored to adhere to the most rigorous methodology in both conducting the systematic reviews that formed the evidentiary basis of the guideline and developing the guideline document. This methodology and summary of the protocols are described in accompanying manuscripts by Tetreault et al within this Focus Issue.^9,10^ In summary, with the funding support of AO Spine and the Praxis Spinal Cord Institute, a Systematic Review Committee and a Guideline Development Group (GDG) for each of the 3 topics was established, which consisted of a wide spectrum of relevant stakeholders, including those with lived experience and clinicians aside from spine surgeons, such as neurologists, rehabilitation specialists, and intensive care physicians. Formal systematic reviews were conducted, adhering to the framework and principles outlined in the AMSTAR 2 critical appraisal tool^11^ and were supported by professional methodologists from Aggregate Analytics (Fircrest, WA, USA; https://www.linkedin.com/company/aggregate-analytics). The GDG then developed recommendations using the Grades of Recommendation, Assessment, Development, and Evaluation (GRADE) framework.^12^ Consensus meetings were held on Zoom with live, anonymous voting in order to arrive at the final recommendations. Important within the GRADE approach is that an evidence-based recommendation is not based solely upon the strength of the published literature, but also considers factors such as feasibility and acceptability of treatment options, cost effectiveness, impact on health inequities, the balance of desirable and adverse effects, as well as patient preferences. For many of these factors (eg, cost-effectiveness) where evidence was limited or unavailable, the role of expert opinion and perspective from individuals with lived experience was documented. Voting on these factors and the related decisions for recommendations was conducted anonymously. Leveraging the widespread familiarity of Zoom video-conferencing stemming from the COVID pandemic, this online meeting technology allowed for inclusion of GDG members from around the globe and facilitated great diversity in the stakeholders represented within the GDGs of the consensus meetings.

### The Timing of Surgical Decompression After Acute Traumatic SCI

One of the most urgent decisions to make when a patient presents to the hospital with an acute traumatic SCI and ongoing cord compression is how urgently to do the surgical decompression. The STASCIS trial reported by Fehlings et al in 2012 was a prospective observational study that revealed that patients decompressed within 24 hours of injury (“early surgery”) had a greater likelihood of achieving 2 or 3 grades of improvement on the ASIA Impairment Scale compared to those decompressed after this time window.^13^ This was arguably the strongest evidence considered in the 2017 AO Spine Guideline which *“suggested that early surgical decompression be considered as a treatment option”* for adult patients with acute SCI (including central cord injuries).^6^ The quality of the evidence was considered low and the use of the wording “suggested” implies a “weak recommendation”. One of the limitations of the evidence at that time was the lack of a meta-analysis due to the heterogeneity of studies exploring the impact of the timing of surgical decompression on neurological recovery. Such a meta-analysis was undertaken by Fehlings and colleagues and reported on in 2021.^14^ This and other available evidence provided a compelling rationale to update the 2017 AO Spine Guideline, as such guidelines (including the ones we are presenting herein) are ‘living documents’ and require re-consideration when new evidence becomes available. Importantly, such evidence may be sought as the result of knowledge gaps identified in the systematic reviews and the guideline itself. This was certainly the case here, where the lack of a meta-analysis (and its impact on the 2017 guideline) inspired efforts to conduct additional studies and such a meta-analysis. With the available evidence, the GDG concluded that early surgical decompression (within 24 hours) be recommended as a treatment option, which reflects a stronger recommendation than what was generated in the 2017 guideline. While it is entirely plausible that there could be neurologic benefits to “ultra-early” surgical decompression done even sooner than 24 hours (eg 8 hours, 12 hours post-injury), the GDG could not make a recommendation about this given the available literature. This is described in detail in the guideline manuscript by Fehlings et al.^15^ In essence, the accrued evidence strongly points to the notion that many have believed for quite some time: that there is indeed a neurological benefit to decompressing the spinal cord in an “early” fashion.

### The Hemodynamic Management of Acute Traumatic SCI

Aside from surgical decompression, augmenting spinal cord perfusion to limit secondary ischemic damage may be one of the only other “neuroprotective” approaches available to clinicians. One of the earliest guidelines provided by the Consortium on Spinal Cord Medicine in 2008 recommended that MAP be maintained at 85-90 mmHg for 7 days,^16^ guidance that was supported by the 2013 AANS/CNS guidelines that recommended MAP be “maintained between 85 and 90 mmHg for 7 days”.^7^ The findings of Vale et al^17^ and Levi et al^18^ from the 1990s provided important substantiation for these recommendations, even though the actual target MAP of 85 mmHg and duration of MAP support were not justified but picked essentially arbitrarily, as admitted by Vale et al in the discussion of their paper.^17^ Given that over a decade of literature has been published since the 2013 guidelines, we felt that a revisiting of this guideline was warranted. Furthermore, an emerging body of work has been published on spinal cord perfusion pressure (SCPP) from Papadopoulos and colleagues^19^ as well as Kwon and colleagues^20^, and we felt that this was worth evaluating within the framework of guideline development (ultimately, we chose not to generate a guideline on SCPP management). Importantly, by doing a systematic review of the literature on MAP augmentation after SCI, it was evident that the current body of literature did not provide strong evidence that MAP augmentation at a particular MAP target improves neurological benefit. To some extent, this lack of certainty weighed heavily in our new guideline, insofar as the lack of high-quality evidence to substantiate a specific MAP threshold made it hard to be very dogmatic about what the MAP target ought to be. The limitations of the literature are highlighted in our accompanying systematic review by Evaniew et al^21^ and summarized in the guideline document by Kwon et al.^22^ What ultimately emerged from this was a new guideline recommendation that suggests a MAP target with a lower level of 75-80 mmHg and an upper limit of 90-95 mmHg, for a period of 3-7 days.

### Intra-Operative Spinal Cord Injury

Given the dreaded nature of intraoperative SCI, we endeavored to provide clinicians guidance around what to do when such a neurologic decline occurs. It is recognized that previous systematic reviews and guidelines have been undertaken, such as those by Hadley et al,^23^ which have confirmed the accuracy of EP monitoring. However, these reviews have acknowledged the lack of a clear benefit of EP monitoring in terms of improving patient outcomes. This work confirms the need for developing care pathways to prevent diagnose and manage intra-operative changes in electrophysiologic monitoring signals. In reviewing this literature, it was evident that the topic itself required a slightly different approach, starting with how to define an “intra-operative SCI”. Hence, the effort was divided into 3 parts: defining “intra-operative SCI”, characterizing its incidence and risk factors, reviewing the diagnostic accuracy of various intra-operative neurophysiologic monitoring techniques, and proposing a care pathway that surgeons may follow when faced with these situations.^24-27^ Firstly, by considering neurological impairment that occurs around surgery and defining it as “SCI”, we sought to de-stigmatize what is known to be a relatively rare event and acknowledge that even in the most technically skilled of hands and with the most meticulous technique, such events occur almost inevitably during the course of a spine surgeon’s career (and that its occurrence does not mean that one is a “bad surgeon”). It is recognized that in the management of some pathologies, such as the resection of intramedullary tumors, the spinal cord is invariably going to be “injured” during the procedure and may result in a worsening of neurological function. Again, we felt that by defining such instances of neurological decline as an “intra-operative SCI”, such inevitable occurrences would be de-stigmatized. Aside from defining this term, we have quantitatively established the diagnostic accuracy of intra-operative neuro-monitoring (IONM) and have developed a recommendation that IONM be used in high-risk spine surgery cases.^24^ And finally, we made a recommendation around different responses as well as treatment approaches and created a checklist and care pathway that surgeons can initiate when monitoring alerts indicate the potential for an ISCI. The GDG, on considering the available evidence, developed 2 guideline statements. The first recommended that IONM be used in patients deemed to be at high risk for an intraoperative SCI — this applies particularly to individuals undergoing surgery for an intramedullary spinal cord tumour, complex spinal deformity and in cases of severe cord compression with myelopathy. The second guideline statement suggested that patients be screened for risk for an intraoperative SCI and that high-risk patients be discussed in a multidisciplinary team fashion and that a care pathway be used for the intraoperative management.

### Conclusion

This Focus Issue provides a snapshot of the existing evidence on these important SCI issues, and a methodologically rigorous framework for developing guidelines (when possible) to help clinicians in their decision-making. Obviously, evidence will continue to accrue, and this exercise of developing guidelines often points to important knowledge gaps which, once filled, may lead to a re-visiting of the guidelines themselves. Such was the case, for example, with the AO Spine Guideline on timing of surgical decompression from 2017.^6^ For hemodynamic management, there are clearly many questions that remain regarding the optimal target and duration of MAP augmentation, and we did not even attempt to establish a guideline for spinal cord perfusion pressure management given the state of this body of literature. And for intra-operative SCI, there is obviously the need to examine how varying treatment approaches actually mitigate the neurologic impairment. We conclude the Focus Issue with a manuscript by Fehlings et al^28^ which describes some of these knowledge gaps and directions that would be rationally taken to fill them in the future. These current guidelines will serve as a ‘living document’ and it is our expectation (and sincere hope) that they will at some point require revision as new, higher quality evidence emerges. Until then, we hope that they will help clinicians in all jurisdictions and health care environments in their efforts to provide the best care possible to SCI patients who have suffered this devastating injury.
